# Recurrent Spontaneous Coronary Dissection in Puerperium Precipitating in Cardiogenic Shock Prompted Coronary Artery Bypass Grafting

**DOI:** 10.1002/ccr3.71028

**Published:** 2026-01-17

**Authors:** Lorenzo Giovannico, Giuseppe Fischetti, Domenico Parigino, Federica Mazzone, Luca Savino, Emanuela De Cillis, Aldo Domenico Milano, Tomaso Bottio

**Affiliations:** ^1^ Dipartimento di Medicina di Precisione e Rigenerativa e Area Jonica Università degli Studi di Bari Aldo Moro Bari Italy

**Keywords:** cardiogenic shock, coronary artery bypass grafting (CABG), postpartum cardiovascular complications, pregnancy‐associated acute coronary syndrome, spontaneous coronary artery dissection (SCAD)

## Abstract

This case report details a rare instance of recurrent spontaneous coronary artery dissection (SCAD) in a 39‐year‐old woman following the delivery of twins, leading to cardiogenic shock. SCAD, although occurring most commonly in middle‐aged women (45–52 years), can also occur in young, otherwise healthy women, in association with pregnancy. In this situation, it poses significant diagnostic and therapeutic challenges, particularly during pregnancy or postpartum periods. This patient's emergency coronary angiography revealed double‐vessel SCAD, and initial treatment with a drug‐eluting stent failed. Surgical intervention with coronary artery bypass grafting (CABG) was ultimately necessary due to recurrent dissection and hemodynamic instability. The case underscores the utility of CABG as a definitive treatment for SCAD, particularly when percutaneous interventions are insufficient. Additionally, the report discusses the importance of early diagnosis using advanced imaging techniques and highlights postoperative management, including beta‐blockers to reduce recurrence risk. This case provides insights into SCAD's complex pathophysiology and the critical role of tailored intervention strategies for young women postpartum.

## Graphical abstract

Graphical summary of postpartum SCAD: (A) ECG with ST‐elevation; (B) surgical revascularization with BIMA and saphenous vein grafts; (C–D) coronary angiography showing RCA and LAD dissection; (E–F) intraoperative subepicardial hematoma of LM and LCx.

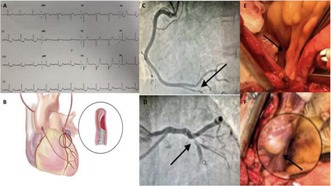

**Summary**

- Recurrent spontaneous coronary artery dissection (SCAD) in the postpartum period may cause life‐threatening cardiogenic shock.
- When percutaneous coronary intervention fails, coronary artery bypass grafting (CABG) can be a lifesaving option in hemodynamically unstable patients.
- Early recognition and tailored management are essential for optimal outcomes in young postpartum women.

## 1 Introduction

SCAD has historically been considered a rare and unfathomed occurrence, typical of young women without traditional risk factors or underlying cardiac conditions. Current evidence challenges this narrative by depicting a prevalence of up to 4% of all ACS [1]. SCAD may originate from an intimal tear with dissection of the medial layer or from an intra‐medial hemorrhage due to rupture of the vasa vasorum [2], which may be progressive and involve the entire coronary district. We present a case of a 39‐year‐old Caucasian woman who was admitted to the emergency room 10 days after a twin vaginal delivery, complaining of chest pain associated with sudden shortness of breath. Emergency coronary angiography was performed, displaying evidence of a double‐vessel SCAD involving the left anterior descending artery and the posterior descending artery. In our case, after the failure of percutaneous treatment, we decided to perform a surgical revascularization. SCAD represents a challenging cause of ACS, affecting young women during pregnancy and puerperium. Treatment is based on the clinical presentation, symptoms, and extension of coronary involvement. The case described herein indicates that CABG is an effective solution in cases of recurrent SCAD.

## 2 Case Report

**FIGURE 1:**
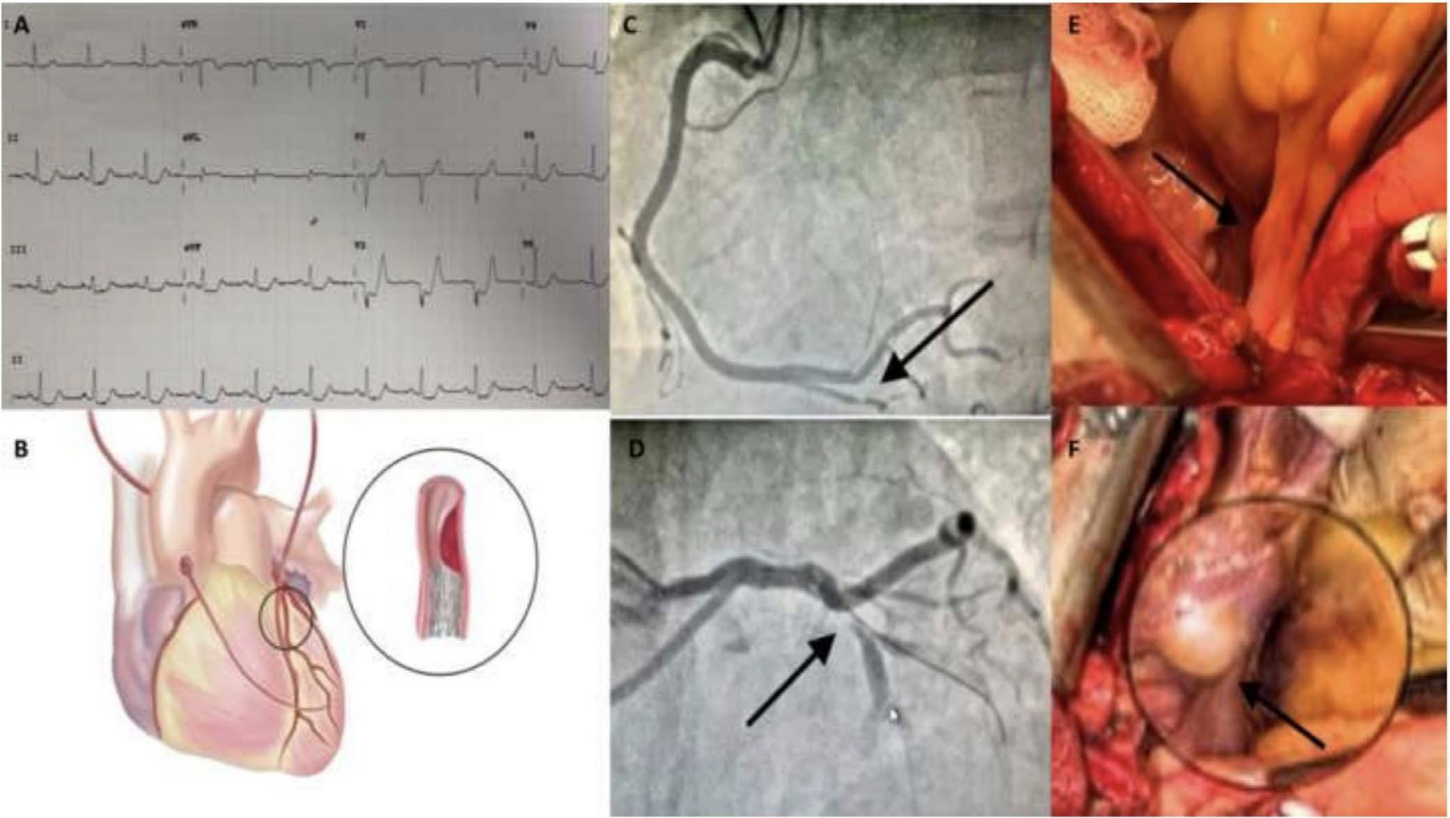
(A) the initial ECG showed ST‐elevations in V3–V4; (B) Left IMA to LAD graft, right IMA to obtuse marginal branch graft, autologous saphenous vein to first diagonal branch graft; LAD segment with DES and SCAD progression; (C) CAG shows dissection of the middle‐distal tract of RCA and (D) ostial dissection of LAD; (E) Subepicardial hematoma of LM and (F) LCx.

A 39‐year‐old Caucasian woman (gravida 1 para 1) was admitted to the emergency room 10 days after a twin vaginal delivery complaining of chest pain associated with sudden shortness of breath. Vital signs were: blood pressure 80/60 mmHg, heart rate 120 bpm, oxygen saturation 92% on room air, arterial pH 7.28, and arterial blood lactate level was 3.3 mmol/L (reference < 2.0 mmol/L). Urine output was preserved at 0.5 mL/kg/h. High‐sensitivity troponin I (hs‐cTnI) was 8750 ng/L (reference < 15 ng/L for women). Electrocardiographic (ECG) showed ST‐elevations in V3–V4 (Figure 1A). Moreover, echocardiography demonstrated reduced left ventricular function (ejection fraction (EF) of 40%). Acute coronary syndrome (ACS) was diagnosed. Emergency coronary angiography (CAG) was performed, displaying evidence of a double‐vessel SCAD involving the left anterior descending artery (LAD) and the posterior descending artery (RCA) (Figure 1C,D). Given the persistence of symptoms and the hemodynamic instability, a primary percutaneous coronary intervention (PCI) approach was chosen, and a drug‐eluting stent (DES) was implanted at the proximal LAD. Post‐PCI, the patient's blood pressure stabilized at 105/70 mmHg, oxygen saturation improved to 96%, and TIMI flow grade was 2 in the LAD. Peak hs‐cTnI reached 9872 ng/L. Due to the good clinical picture of the patient, it was decided not to perform a second coronary angiogram. On the third postoperative day, the high‐sensitivity troponin level decreased to 2500 ng/L. On postoperative day 4, the patient experienced recurrent chest pain and acute dyspnea with rapidly worsening hemodynamics: blood pressure dropped to 80/55 mmHg, heart rate 130 bpm, oxygen saturation decreased to 89% on room air, and arterial pH fell to 7.20. Urine output declined to 0.3 mL/kg/h. ECG again showed ST‐segment elevation in V3–V4, and echocardiography revealed a markedly reduced EF of 20%. In light of these findings and suspected recurrent dissection, urgent surgical myocardial revascularization was indicated.

Urgent on‐pump surgical myocardial revascularization was performed. During the operation, a subepicardial hematoma over the left main (LM) and left circumflex arteries (LCx) was found, showing the progression of SCAD to these segments (Figure 1E,F). Bilateral internal mammary artery (BIMA) was grafted to the distal LAD and first obtuse marginal branch, followed by a segment of the autologous saphenous vein that was grafted onto the first diagonal branch (Figure 1B). The postoperative course was uneventful, with progressive stabilization of hemodynamics and normalization of oxygenation and diuresis. At 1‐year follow‐up, echocardiography showed complete recovery of left ventricular function with EF of 60%, and the patient remained free of recurrent cardiovascular events.

## 3 Differential Diagnosis, Investigations and Treatment

Differential diagnosis:

- Acute Coronary Syndrome
- Pulmonary Embolism
- Peripartum Cardiomyopathy

Investigations:

- ECG: ST‐elevations in V3–V4
- Troponin‐I: Elevated
- Echocardiography: EF 40% initially, dropped to 20% post‐recurrence
- Coronary Angiography: Double‐vessel SCAD (LAD and RCA)

Treatment

- Initial percutaneous intervention with DES implantation in LAD.
- Recurrence of SCAD led to emergency CABG using BIMA and saphenous vein grafts.

## 4 Conclusion and Results

SCAD is an uncommon but serious cause of ACS, particularly relevant in pregnancy and puerperium. Management depends on hemodynamic stability and the extent of coronary involvement. In our case, urgent CABG was effective after PCI failure, underscoring that surgical revascularization remains a valuable option in recurrent SCAD with severe hemodynamic compromise.

## 5 Discussion

SCAD has historically been considered a rare and unfathomed occurrence, typical of young women without any apparent cardiac condition [1]. Our case provides insights that warrant further investigation. First, the augmented occurrence of SCAD in pregnancy and puerperium is largely assessed, as is its association with a jeopardized outcome, whereas its pathogenesis remains controversial [2]. During gestation, renowned hemodynamic changes occur: a physiological cardiac demand represented by a hypervolemic and hyperdynamic state. In addition, perhaps because of a peculiar hormonal layout, structural modifications occur in the arterial wall, which adds to the vascular strains of labor and delivery, all resulting in a weakened coronary intimal integrity with the consequent risk of tears [2]. Here, we present the case of a healthy woman who experienced twin vaginal delivery, an event perceived as a gargantuan physical and psychological stressor, presenting with SCAD in the puerperium. In our case, the pregnancy was natural without the use of assisted reproductive techniques. It is important to underline this because patients with SCAD were more frequently treated for subfertility (28% versus 12%). Of the pregnant women with SCAD, 9% had undergone IVF. Women with SCAD in pregnancy were also more likely to have a history of single or combination subfertility treatment (28% versus 16%) [2].

SCAD may originate from an intimal tear with dissection of the medial layer or from an intra‐medial hemorrhage due to rupture of the vasa vasorum [2], which may be progressive and involve the entire coronary district, as in our case, where SCAD was initially limited and then extended to other branches. Interestingly, postmortem examinations of pregnant women reported eosinophilic infiltrates within the adventitia, although it is unclear whether they represent the harmful effect of SCAD through a lytic mechanism or whether they trivially are a consequence of this event [2]. Another insight from our case is the need for immediate recognition of SCAD, which can deviate from atypical symptoms and unspecific ECG. The use of CAG in this scenario is indisputable; however, such a 2D angiogram can efficiently assess luminal narrowing but has poor sensitivity for wall abnormalities that are coherently pathognomonic for SCAD [3, 4]. Indeed, on post‐operative day 4, abrupt symptomatology accompanied by hemodynamic instability, sharp troponin increase, and strongly depressed EF led to the diagnosis of recurrent SCAD. The treatment of choice also deserves some analysis; performing a cardio‐pulmonary bypass‐assisted coronary artery bypass graft (CABG) in the puerperium does not represent a banal procedure. In fact, the hypervolemic and hyperdynamic state, further psychophysical stress to the patient, and vascular damage overlapping with the immunoinflammatory and hemostatic derangements associated with surgery and cardio‐pulmonary bypass are factors to consider in the risk–benefit assessment. A scenario made it even more convolutely challenging in the case of a current pregnancy. Although SCAD is rare, it does occur in young women; it is mandatory to guarantee long‐term outcomes using reliable surgical techniques. In this sense, patients with recurrent SCAD undergoing CABG would benefit from a total arterial approach. In the present case, we harvested the bilateral IMAs and performed a saphenous vein stand‐alone graft on the first diagonal branch to avoid plausible complications of sequential grafting, such as impaired long‐term patency. CABG still represents the gold standard for recurrent SCAD with hemodynamic compromise because it allows effective revascularization, as demonstrated in the present case. In fact, a 1‐year follow‐up revealed complete recovery of myocardial function with non‐significant anterior wall hypokinesia. The last insight from our case is related to the importance of postoperative management of hypertension (i.e., associated with SCAD recurrence) with beta‐blockers, which have been shown to reduce recurrent SCAD by almost two‐thirds [1].

## Author Contributions

**Lorenzo Giovannico:** validation, visualization, writing – original draft, writing – review and editing. **Giuseppe Fischetti:** validation, visualization, writing – review and editing. **Domenico Parigino:** validation, visualization, writing – review and editing. **Federica Mazzone:** visualization, writing – review and editing. **Luca Savino:** validation, writing – review and editing. **Emanuela De Cillis:** validation, visualization, writing – review and editing. **Aldo Domenico Milano:** validation, visualization, writing – review and editing. **Tomaso Bottio:** supervision, validation, visualization, writing – original draft, writing – review and editing.

## Consent

The authors should confirm that written informed consent was obtained from the patient to publish this report in accordance with the journal's patient consent policy.

## Conflicts of Interest

The authors declare no conflicts of interest.

## Data Availability

The data supporting the findings of this study are available from the corresponding author [LG] upon reasonable request. The data are not publicly available due to privacy restrictions.
